# Daily Energy and Protein Intake Requirements to Prevent Body Weight Loss in Elderly Residents of Nursing Homes

**DOI:** 10.7759/cureus.83983

**Published:** 2025-05-12

**Authors:** Sonoko Yasui-Yamada, Teruhiro Morishita, Nobuhiko Umemoto, Rie Tsutsumi, Kazuyo Kohno, Shunsuke Shiota, Mina Shinohara, Yuna Izumi-Mishima, Kazuhiro Nomura, Hiroshi Sakaue, Eiji Takeda

**Affiliations:** 1 Department of Nutrition and Metabolism, Institute of Biomedical Sciences, Tokushima University Graduate School, Tokushima, JPN; 2 Department of Physical Therapy, Kenshokai Gakuen College for Health and Welfare, Tokushima, JPN; 3 Nursing Home for Aged Persons, Kenshokai Group, Mima, JPN; 4 Department of Care Welfare, Kenshokai Gakuen College for Health and Welfare, Tokushima, JPN

**Keywords:** adults aged 75 and over, body weight, elderly, energy intake, nursing home residents, protein intake, weight loss

## Abstract

Background

Maintaining body weight is crucial for preserving the health of older adults. This study aimed to identify the daily energy and protein intake levels necessary to prevent body weight loss exceeding 1 kg over a six-month period among nursing home residents.

Methodology

A retrospective analysis was conducted on 101 residents aged ≥75 years. Data on body weight changes and dietary intake over a continuous six-month period were reviewed. The association between dietary intake and body weight loss greater than 1 kg was evaluated in 78 female residents.

Results

Among the 101 residents (23 males and 78 females; median age: 91 years (interquartile range (IQR) = 86-94); median body mass index (BMI): 21.3 kg/m² (IQR = 18.7-23.7)), 41% experienced a body weight loss of more than 1 kg over six months, despite receiving a median energy intake of 24.7 kcal/kg/day (IQR = 19.6-29.2) and a protein intake of 0.98 g/kg/day (IQR = 0.81-1.20). Multivariate logistic regression analysis adjusted for age and BMI in female residents revealed that an energy intake exceeding 30 kcal/kg/day was significantly associated with a reduced risk of body weight loss (odds ratio (OR) = 0.17, 95% confidence interval (CI) 0.04-0.68, p = 0.013), as was protein intake exceeding 1.2 g/kg/day (OR = 0.12, 95% CI = 0.03-0.49, p = 0.003).

Conclusions

To prevent body weight loss in elderly female nursing home residents, a daily intake of at least 30 kcal/kg/day of energy and 1.2 g/kg/day of protein is recommended. These findings highlight the importance of proactive nutritional management in long-term care facilities to support better health outcomes in this vulnerable population.

## Introduction

Aging is significantly characterized by changes in body composition and muscle loss, which result from altered dietary intake and physical activity, the presence of multiple chronic diseases, hormonal changes, increased susceptibility to acute illnesses, and progressive body weight (BW) loss [1,2]. From the age of 70 years onwards, both lean body mass and total BW decrease [3]. Compared with younger individuals, most of the tissue lost during weight loss in the elderly is lean tissue, mainly skeletal muscle. Excessive loss of lean tissue leads to sarcopenia and deterioration in overall health status. Sarcopenia is associated with a high risk of a wide range of adverse health outcomes, such as falls, functional decline, physical disability, frailty, cognitive impairment, metabolic disorders, and mortality [4]. Older age may be the most important risk factor for sarcopenia, but poor nutritional status is also independently associated with sarcopenia [5]. Therefore, maintaining adequate dietary intake and nutritional status is essential for health determinants in the elderly population [6,7].

The number of nursing home residents has been increasing worldwide. In the United States, the number of residents was estimated at approximately 1.6 million in 2015, and in Europe, about 4.0 million in 2017 [8]. Japan is one of the most rapidly aging countries. According to a survey by the Ministry of Health, Labour and Welfare, the number of nursing care facility beds in Japan has been increasing, reaching approximately 1.0 million in 2023 [9]. Malnutrition is a common issue among older adults in nursing homes, with prevalence reported as high as 12-54% [10] and 52-85% [11]. Malnutrition is associated with adverse health outcomes, including increased risk of falls, impaired immune function, reduced quality of life, prolonged and more frequent hospitalizations, and, ultimately, higher mortality [12-15]. Although assessing body composition, particularly muscle mass, is ideal for evaluating malnutrition, such assessments are often difficult to perform in nursing homes due to limited access to appropriate tools. In a study focusing on BW, Wirth et al. reported that a low body mass index (BMI <20 kg/m^2^) and weight loss of 5 kg or more were important factors associated with six-month mortality [16]. These findings suggest the importance of maintaining BW in long-term care facility residents to support their health and longevity.

Therefore, the aim of this study was to determine the daily energy and protein intake requirements necessary to prevent BW loss in nursing home residents.

## Materials and methods

Study design and participants

This was a retrospective, observational, single-center study. The study was conducted at a nursing home with a total of 100 residents. The comprehensive team consists of physicians, care managers, care workers, nurses, physical therapists, occupational therapists, and a registered dietitian. The registered dietitian provides individual nutritional care, including the provision of appropriate food types and quantities, nutritional counseling, and monitoring of dietary intake and nutritional status to ensure effective and personalized nutritional support for each resident. Participants were selected from among residents living in the facility between 2022 and 2023. Inclusion criteria were age ≥75 years and availability of documented data on BW and dietary intake over a continuous six-month period. A total of 101 elderly residents (23 males and 78 females) met these criteria and agreed to participate in the study. This study was conducted in accordance with the principles of the Declaration of Helsinki. The study protocol was approved by the Ethics Committee of the Tokushima University Hospital (approval number: 4553).

Data collection

The following characteristics were obtained from the residents’ care records: sex, age, BW, body height, dietary intake, medication status, and comorbidities. Comorbidities were categorized as metabolic, circulatory, pulmonary, gastrointestinal, renal and urological, orthopedic, neurological, psychological, and other diseases. Data on food intake percentages were obtained from care records, and nutritional intake was estimated by multiplying the amount of food provided by the facility by these percentages. Dietary intake was expressed as energy intake (kcal/kg/day) and protein intake (g/kg/day). All participants consumed food orally; none received enteral (nasogastric tube) or parenteral (intravenous) nutrition.

BW was assessed monthly by nursing staff and caregivers using calibrated wheelchair-accessible scales. Measurements were performed with participants wearing light clothing and no shoes. BMI was calculated by dividing the BW by the height squared (kg/m^2^). In this study, weight loss was defined as a reduction of 1 kg or more over a six-month period.

Statistical analyses

Categorical variables were presented as numbers and percentages, while continuous variables were expressed as median and interquartile range (IQR). Continuous variables were compared using the Wilcoxon rank-sum test. Categorical variables were compared using the chi-squared test. Univariate and multivariate logistic regression analyses were conducted to calculate odds ratios (ORs) and 95% confidence intervals (CIs) to evaluate the association between dietary intake and BW loss of more than 1 kg over a six-month period. Multivariate analyses were performed with adjustments for age and BMI. All statistical analyses were performed using JMP ver 13 software (SAS Institute, Cary, NC, USA). A p-value <0.05 was considered statistically significant.

## Results

Characteristics of the participants

Table 1 shows the characteristics of the study participants. A total of 101 residents (23 males, 78 females; median age: 91 years (IQR = 86-94)) were included in the study. Among them, 9% (9/101) were aged 75-80 years, 35% (35/101) were aged 80-90 years, and 56% (57/101) were over 90 years of age. The median baseline BMI was 21.3 kg/m² (IQR = 18.7-23.7). According to the Dietary Reference Intakes for Japanese, 2025 [17], 53% (53/101) of the participants were classified as underweight (BMI < 21.5 kg/m²), 32% (32/101) as normal weight (BMI = 21.5-25 kg/m²), and 16% (16/101) as overweight (BMI ≥ 25 kg/m²). The median energy intake over the six-month period was 24.7 kcal/kg/day (IQR = 19.6-29.2), and the median protein intake was 0.98 g/kg/day (IQR = 0.81-1.20). A total of 29 residents (4 males and 25 females) used oral nutritional supplements. During the period, 41% (41/101) of participants experienced a BW loss of 1 kg or more.

**Table 1:** Characteristics of the participants. Continuous variables were compared using the Wilcoxon rank-sum test. Categorical variables were compared using the chi-squared test. BW: body weight; BMI: body mass index

	All (n = 101)	Male (n = 23)	Female (n = 78)	P-value	Test statistic
Age (years)	91 (86-94)	87 (82-90)	91 (88-94)	0.001	z = −3.22
75 ≤ Age < 80, n (%)	9 (9)	5 (22)	4 (5)	0.013	χ² = 8.64
80 ≤ Age < 90, n (%)	35 (35)	10 (43)	25 (32)		
90 ≤ Age, n (%)	57 (56)	8 (35)	49 (63)		
Height (cm)	145.0 (138.0-150.6)	154.3 (148.5-163.0)	141.8 (137.0-147.0)	<0.001	z = 5.44
Baseline BW (kg)	44.6 (37.4-51.4)	53.1 (44.6-60.4)	42.2 (36.2-47.6)	<0.001	z = 4.43
Baseline BMI (kg/m²)	21.3 (18.7-23.7)	22.9 (19.0-24.5)	20.9 (18.6-23.3)	0.063	z = 1.86
BMI < 21.5, n (%)	53 (52)	8 (35)	45 (58)	0.154	χ² = 3.74
21.5 ≤ BMI < 25, n (%)	32 (32)	10 (44)	22 (28)		
25 ≤ BMI, n (%)	16 (16)	5 (22)	11 (14)		
Comorbidities					
Metabolic diseases	20 (20)	3 (13)	17 (22)	0.355	χ² = 0.86
Circulatory diseases	51 (50)	9 (39)	42 (54)	0.215	χ² = 1.54
Pulmonary diseases	8 (8)	1 (4)	7 (9)	0.470	χ² = 0.52
Gastrointestinal diseases	16 (16)	1 (4)	15 (19)	0.086	χ² = 2.95
Renal and urological diseases	9 (9)	3 (13)	6 (8)	0.429	χ² = 0.63
Orthopedic diseases	37 (37)	2 (9)	35 (45)	0.002	χ² = 10.0
Neurological diseases	37 (37)	7 (30)	30 (38)	0.483	χ² = 0.49
Psychological diseases	6 (6)	0 (0)	6 (8)	0.170	χ² = 1.88
Other diseases	27 (27)	4 (17)	23 (29)	0.249	χ² = 1.33
BW change for 6 months (kg)	−0.6 (−2.4-1.0)	−0.9 (−2.7-0.5)	−0.3 (−2.2-1.0)	0.335	z = −0.96
BW loss ≥ 1 kg, n (%)	41 (41)	11 (48)	30 (38)	0.422	χ² = 0.65
BW loss < 1 kg, n (%)	60 (59)	12 (52)	48 (62)		
Daily energy intake for 6 months (kcal/kg/day)	24.7 (19.6-29.2)	23.8 (19.2-28.7)	24.8 (20.1-30.1)	0.500	z = −0.68
Daily protein intake for 6 months (g/kg/day)	0.98 (0.81-1.20)	0.95 (0.78-1.14)	1.03 (0.84-1.23)	0.311	z = −1.01

Association between daily energy and protein intake and BW loss in females

Due to the limited number of male participants, the analysis of the association between nutrient intake and BW loss was conducted only among female participants. Univariate logistic regression analysis demonstrated significant associations between BW loss (≥1 kg) and both energy intake (OR = 0.89, 95% CI = 0.82-0.97; p = 0.002) and protein intake (OR = 0.10, 95% CI = 0.02-0.63; p = 0.009). No significant associations were observed with age or BMI (Table 2, univariate models). In multivariate logistic regression analysis adjusted for age and BMI, both energy intake (OR = 0.87, 95% CI = 0.79-0.95, p < 0.001) and protein intake (OR = 0.08, 95% CI = 0.01-0.55, p = 0.006) remained significantly associated with the risk of BW loss (Table 2, multivariate models 1 and 2).

**Table 2:** Univariate and multivariate analyses of risk factors associated with BW loss ≥1 kg in females. Univariate and multivariate logistic regression analyses were conducted to calculate ORs and 95% CIs among female residents (n = 78). Multivariate analyses were performed with adjustments for age and BMI. Model 1 included daily energy intake as an independent variable, while Model 2 included daily protein intake. OR: odds ratio; CI: confidence interval; BMI: body mass index

	Univariate			Multivariate 1			Multivariate 2		
	OR	95% CI	P-value	OR	95% CI	P-value	OR	95% CI	P-value
Age (years)	1.03	0.95-1.11	0.510	1.01	0.92-1.10	0.870	1.02	0.94-1.11	0.579
BMI (kg/m²)	0.99	0.88-1.11	0.873	0.90	0.79-1.03	0.129	0.94	0.83-1.07	0.339
Energy intake (kcal/kg/day)	0.89	0.82-0.97	0.002	0.87	0.79-0.95	<0.001	-	-	-
Protein intake (g/kg/day)	0.10	0.02-0.63	0.009	-	-	-	0.08	0.01-0.55	0.006

Further analysis revealed that an energy intake exceeding 30 kcal/kg/day was significantly associated with a reduced risk of BW loss (OR = 0.17, 95% CI = 0.04-0.68, p = 0.013), as was protein intake exceeding 1.2 g/kg/day (OR = 0.12, 95% CI = 0.03-0.49, p = 0.003) (Table 3, multivariate models 2 and 4). In contrast, intakes of 25 kcal/kg/day and 1.0 g/kg/day of protein did not demonstrate a significant protective effect against BW loss (Table 3, multivariate models 1 and 3).

**Table 3:** Multivariate analyses of risk factors associated with BW loss ≥1 kg in females. Multivariate logistic regression analyses were conducted to calculate ORs and 95% CIs, adjusted for age and BMI among female residents (n = 78). Models 1 and 2 included daily energy intake as an independent variable, while Models 3 and 4 included daily protein intake. OR: odds ratio; CI: confidence interval; BMI: body mass index

	Multivariate 1			Multivariate 2			Multivariate 3			Multivariate 4		
	OR	95% CI	P-value	OR	95% CI	P-value	OR	95% CI	P-value	OR	95% CI	P-value
Age (years)	1.02	0.94-1.11	0.578	1.02	0.94-1.11	0.659	1.03	0.95-1.11	0.504	1.05	0.96-1.14	0.279
BMI (kg/m²)	0.96	0.85-1.09	0.554	0.93	0.82-1.06	0.291	0.97	0.86-1.10	0.681	0.91	0.80-1.05	0.174
Energy intake												
≥25 kcal/kg/day	0.53	0.20-1.41	0.202	-	-	-	-	-	-	-	-	-
≥30 kcal/kg/day	-	-	-	0.17	0.04-0.68	0.013	-	-	-	-	-	-
Protein intake												
≥1.0 g/kg/day	-	-	-	-	-	-	0.70	0.27-1.84	0.469	-	-	-
≥1.2 g/kg/day	-	-	-	-	-	-	-	-	-	0.12	0.03-0.49	0.003

## Discussion

In this study, 41% (41/101) of elderly Japanese nursing home residents (23 males and 78 females; median age = 91 years (IQR = 86-94)) who received a median intake of 24.7 kcal/kg/day (IQR = 19.6-29.2) of energy and 0.98 g/kg/day (IQR = 0.81-1.20) of protein experienced a BW loss of more than 1 kg over a six-month period (Table 1). At baseline, the median BMI was 21.3 kg/m^2^ (IQR = 18.7-23.7). According to the Japanese classification of BW status, 53% (53/101) were underweight (BMI < 21.5 kg/m^2^), 32% (32/101) had a normal weight (BMI 21.5-25 kg/m^2^), and 15.8% (16/101) were overweight (BMI ≥ 25 kg/m^2^). Our findings suggest that a daily intake exceeding 30 kcal/kg of energy and 1.2 g/kg of protein may help prevent weight loss, while intakes of 25 kcal/kg of energy and 1.0 g/kg of protein were insufficient in elderly female residents.

It is well-established that weight loss in older adults results in a disproportionately greater loss of lean body mass compared to younger individuals. Malnutrition among the elderly is a common concern and has been associated with multiple adverse outcomes, including decreased quality of life, increased susceptibility to medical complications, higher rates of hospitalization, and elevated mortality [18,19]. Alarmingly, malnutrition remains prevalent even in developed countries. Studies have estimated that up to 15% of community-dwelling and home-bound older adults, 23-62% of hospitalized elderly patients, and up to 85% of nursing home residents suffer from protein-energy malnutrition [20]. Compared with community-dwelling elderly people, nursing home residents generally have lower BMIs and exhibit more frequent and severe nutritional deficiencies. BMI tends to decline with advancing age due to reduced dietary intake and physical activity, the presence of multiple chronic and acute diseases, hormonal alterations, and unintentional weight loss [1,2]. In our study, participants had a notably higher median age (91 years (IQR = 86-94)) than that reported in studies from the Netherlands (85 ± 7.3 years) [21], Canada (84.7 ± 7.7 years) [22], and the European Union (83.6 ± 9.4 years) [23]. Additionally, their median BMI (21.3 kg/m² (IQR = 18.7-23.7)) was significantly lower than the averages reported from the Netherlands (26 ± 5 kg/m²) [21] and the European Union (25.7 kg/m²) [24].

A previous study investigating the association between BW change and mortality among community-dwelling older adults found that individuals in the ≥5% weight loss group had a significantly increased risk of mortality compared to those in the stable weight group, with the elevated risk persisting after multivariate adjustment (hazard ratio = 1.67, 95% CI = 1.29-2.15) over a four-year follow-up period [25]. No increased mortality was observed among individuals who gained weight [25]. In the Systolic Hypertension in the Elderly Program (SHEP) study [26], which included 4,736 individuals aged 60 years or older, the OR for mortality was 4.9 (95% CI = 3.5-6.8) for those who lost ≥1.6 kg per year compared to those with stable weight, after adjusting for baseline BMI and other covariates. Among those with a low baseline BMI (<23.6 kg/m^2^), weight loss ≥1.6 kg per year was associated with a 22.6% mortality rate, nearly 20 times higher than in those with stable weight and a baseline BMI between 23.6 and 28 kg/m^2^. These findings suggest a synergistically negative effect on survival when low baseline BMI is combined with further weight loss in older adults. The optimum BMI for survival in individuals aged 70 years and older appears to lie between 25 and 30 kg/m^2^ [27]. In a cohort of 12,725 individuals aged ≥65 years, total life expectancy was the highest at a BMI between 25-27 kg/m^2^, while disability-free life expectancy peaked at a BMI of 24 kg/m^2^ [28]. Substantial evidence indicates that BMIs below approximately 21-22 kg/m^2^ are associated with adverse health outcomes and increased mortality in the elderly, with BMIs under 18.5 kg/m^2^ being of particular concern [26,28,29].

Additionally, a previous study demonstrated a significant association between ≥5% weight loss over a one-year period and a transition to dysphagia diet among elderly nursing home residents [30]. Other studies have reported that increased mortality in individuals with ≥5% weight loss over a six-month period was primarily caused by disease progression, social factors, psychiatric factors, and aging [31,32]. In our study, weight loss was defined as a ≥1 kg reduction over six months. Although a 5% reduction is commonly used in criteria such as the Global Leadership Initiative on Malnutrition (GLIM) [33], we adopted an absolute threshold of 1 kg, considering our population’s characteristics. Only 27% experienced ≥5% weight loss, which limited the statistical power. In contrast, 41% lost at least 1 kg, providing a more robust basis for multivariable analysis. In our cohort, a 1 kg loss corresponded to approximately 2% of baseline BW, aligning with prior definitions such as the 2% cachexia criterion by Fearon et al. [34].

Adequate energy intake is essential to prevent protein from being utilized as an energy source [35,36]. The estimated energy requirement is approximately 30 kcal/kg/day for general older adults [37], 27 kcal/kg/day for those with low activity levels [37,38], and 32-38 kcal/kg/day for underweight older adults [37,38]. For protein, the recommended daily intake is 0.75-0.83 g/kg/day for healthy older adults [39,40], with recent guidelines suggesting 1.0-1.2 g/kg/day for healthy elderly [37], and 1.2-1.5 g/kg/day for frail individuals [41]. For the Japanese elderly, energy requirements are estimated at 36 kcal/kg/day for the general elderly population, and 30 kcal/kg/day for those with low physical activity [17]. The recommended protein intake is 1.0 g/kg/day for healthy older adults and ≥1.2 g/kg/day to prevent sarcopenia and frailty. The findings of the present study align with these recommendations, demonstrating that lower daily energy and protein intakes were significantly associated with ≥1.0 kg BW loss over six months in female nursing home residents (Tables 2, 3). These results underscore the importance of maintaining adequate nutritional intake to prevent undernutrition and associated health risks in this vulnerable population.

This study has several limitations. First, the association between dietary intake and BW loss was only examined in female participants due to the limited number of male residents, which may limit the generalizability of the findings to both sexes. Second, as an observational study, this research could establish associations but not infer causality between dietary intake and BW loss. Third, the factors that may influence BW changes, such as mobility level, physical activity, and dietary texture, were not assessed, as the retrospective design did not allow for the collection of such data. Fourth, although maintaining not only BW but also muscle mass is important for better health outcomes in the elderly, we were unable to collect information on body composition and therefore could not address this issue in the present study. Future larger-scale prospective studies that include body composition assessments are warranted to clarify the energy and protein intake requirements necessary to prevent losses in BW and muscle mass among both male and female nursing home residents.

## Conclusions

Weight loss and undernutrition in older adults are often linked to poor oral intake; however, their association with adverse health outcomes remains frequently underrecognized. Adequate dietary protein intake is especially crucial in older populations to maintain muscle mass, promote wound healing and skin integrity, support immune function, and facilitate recovery from illness. This study found that many residents are at risk of insufficient nutritional intake. In conclusion, to prevent BW loss in elderly female nursing home residents, an optimal dietary intake should include at least 30 kcal/kg/day of energy and 1.2 g/kg/day of protein. These findings highlight the need for proactive nutritional management in long-term care facilities to promote better health outcomes in this vulnerable population.
